# *CYB5A* polymorphism increases androgens and reduces risk of rheumatoid arthritis in women

**DOI:** 10.1186/s13075-015-0574-9

**Published:** 2015-03-11

**Authors:** Klaus Stark, Rainer H Straub, Jozef Rovenský, Stanislava Blažičková, Gabriele Eiselt, Martin Schmidt

**Affiliations:** Department of Internal Medicine II, University Hospital Regensburg, Regensburg, Germany; Department of Genetic Epidemiology, University Regensburg, Regensburg, Germany; Laboratory of Experimental Rheumatology & Neuroendocrine Immunology, Department of Internal Medicine I, University Hospital Regensburg, BIOPARK 1, Josef-Engert-Straße 9, 93053 Regensburg, Germany; National Institute of Rheumatic Diseases, Piešt’any, Slovakia; Department of Laboratory Medicine, Faculty of Social Work and Health, University of Trnava, Trnava, Slovakia; Institute of Biochemistry II, Jena University Hospital – Friedrich Schiller University Jena, Jena, Germany

## Abstract

**Introduction:**

Rheumatoid arthritis (RA) is characterized by decreased androgen levels, which was the first hormonal abnormality described. Several studies indicated that steroidogenesis is directed towards endogenous glucocorticoids at the expense of androgens. The decisive step governing androgen synthesis is the 17,20-lyase activity of the *CYP17A1* gene-encoded enzyme cytochrome P450 17A1. Here, we focused on the role in RA of the critical cofactor for 17,20-lyase activity, cytochrome b5, encoded by the *CYB5A* gene.

**Methods:**

Data sets of two genome wide RA association studies (GWAS) were screened for single nucleotide polymorphisms (SNP) in the *CYB5A* gene. Candidate SNPs in *CYB5A* were studied in a case–control study population of Slovakia. Expression analyses were done in synovial fibroblasts from RA patients by quantitative real-time polymerase chain reaction, and cytochrome b5–expression was detected by immunohistochemistry. Real-life androgen production after steroid conversion was measured using radiolabeled substrates.

**Results:**

The study identified the RA-associated intronic SNP rs1790834 in the *CYB5A* gene in one GWAS and confirmed the same SNP in our study. The minor allele reduced RA risk selectively in women (P = 4.1*10^−3^; OR = 0.63, 95% CI [0.46-0.86]). The protective effect was confined to rheumatoid factor-positive (OR = 0.53, [0.37-0.75]) and anti-cyclic citrullinated peptide-positive (OR = 0.58, [0.41-0.83]) cases, respectively. The protective allele doubles *CYB5A* mRNA-expression resulting in 2-3fold activation of steroid 17,20-lyase activity, and protective allele was accompanied by a higher density of cytochrome b5-positive cells in synovial tissue.

**Conclusions:**

*CYB5A* is the first RA susceptibility gene involved in androgen synthesis. Our functional analysis of SNP rs1790834 indicates that it contributes to the sex bias observed in RA.

## Introduction

Rheumatoid arthritis (RA) is a chronic inflammatory joint disease that affects about 0.5 to 1.0% of the population. It causes increasing disability leading to a huge socioeconomic burden [1]. In RA, risk variants in 46 loci explain about half of the genetic risk, indicating that other so far unknown loci are involved [2,3]. Despite the autoimmune etiology, neuroendocrine immune pathways relevant for inflammatory processes are discussed in RA onset and progression [4]. In RA there is a clear preponderance of affected women over men (3:1) [5]. This suggests that high concentrations of estrogens, low concentrations of androgens, or a combination of both increase the risk for RA [6].

Several androgens have anti-inflammatory properties. Dehydroepiandrosterone (DHEA), androstenedione, and testosterone inhibit secretion of IL-1β, IL-6, TNF, and others [7-12]. The androgen 5α-dihydrotestosterone inhibits activation of the human IL-6 gene promoter stimulated by nuclear factor kappa B [13], and it decreases T cell proliferation [14]. There is evidence that some RA patients of both sexes have reduced amounts of serum androgens, even years before disease onset [15,16]. Particularly, female RA patients have lower than normal levels of DHEA and/or DHEA sulfate. In male RA patients, levels of serum testosterone are negatively correlated with disease severity [15]. Two double-blind interventional studies with testosterone demonstrated some benefit in patients with RA [17,18]. A preponderance of serum glucocorticoids over serum androgens is known in many chronic inflammatory diseases, which normalizes after anti-TNF therapy [19]. In summary, there is convincing evidence that a relative lack of androgens is involved in the etiology of RA.

However, we do not know molecular mechanisms responsible for this state of androgen deficiency. A recent study revealed a negative correlation of serum testosterone levels with RA disease activity in male patients under therapy with disease-modifying anti-inflammatory drugs [20]. This indicates that the inflammatory disease can reduce androgen levels. This may depend on increased androgen-to-estrogen conversion that can happen in inflammatory cells such as macrophages and osteoblasts [21-23]. Increased estrogen formation and estrogen-to-androgen ratios were found in the synovial fluid of RA patients compared to controls showing increased aromatase activity [24]. We demonstrated that mixed synoviocytes from patients with RA and osteoarthritis convert DHEA, androstenedione, and testosterone into downstream hormones [25]. If androgens like testosterone are missing in the tissue, synovial aromatase activity is strongly stimulated, and this leads to a very high synovial estrogen-to-testosterone ratio [6,25].

In addition, in collagen type-II arthritic animals and in synovial fibroblasts from RA patients, conversion of DHEA into the proinflammatory metabolite 7α hydroxy-DHEA is increased (gene *CYP7B1*) [26,27]. This means that DHEA is not available for conversion to androgens such as androstenedione and testosterone.

However, these findings in patients with overt inflammatory disease do not explain why androgen levels are lower than normal before disease outbreak [15,16]. Some authors speculated that male RA patients without glucocorticoid treatment might be in a state of (compensated) partial gonadal failure [28]. However, molecular pathways of adrenal or gonadal failure are not known, and this was subject of our study.

Endogenous *de novo* synthesis of androgens depends on two key enzyme activities, 17α-hydroxylase and 17,20-lyase, both linked to one protein encoded by the cytochrome P450 17A1 gene *CYP17A1* [29]. While 17α-hydroxylase activity is essential for synthesis of androgens and cortisol depending on the presence of NADPH-cytochrome P450 reductase (POR) [29], the 17,20-lyase activity depends on the combined presence of POR and the cofactor cytochrome b5 type A (gene on chromosome 18, *CYB5A*) [29,30].

In order to study molecular pathways relevant to androgen deficiency, we focused on cytochrome P450 17A1 and cytochrome b5 type A. In the two published RA genome-wide association study (GWAS) datasets, we reanalyzed single nucleotide polymorphisms (SNP) in the *CYB5A* gene [31,32]. In a novel RA case-control study with Slovak people, we investigated the same SNPs in the *CYB5A* gene. Then, we functionally analyzed SNP alleles by steroidogenic gene expression and pregnenolone conversion into androgens in synovial fibroblasts of RA patients. In addition, density of cytochrome b5A-positive synovial cells was investigated in RA synovial tissue.

## Methods

### Study sample

A total of 842 (117 male, 665 female) Slovak individuals were included for genotyping, with 521 (87 male, 434 female) RA patients and 321 (90 male, 231 female) healthy controls without any arthritic symptoms. Rheumatoid factor (RF) was determined by standard techniques. Measurement of antibody against cyclic citrullinated peptide (anti-CCP) was carried out using an anti-CCP ELISA (Euroimmun, Lübeck, Germany) following the manufacturer’s instructions. From a total of 304 RA patients anti-CCP levels were determined. Values <4.2 RU/ml were anti-CCP negative. The characteristics of these patients are given in Table 1.

**Table 1:** **Characteristics of the study sample for association analyses**

**Variable**	**Rheumatoid arthritis cases**	**Rheumatoid arthritis-free controls**
	**(n = 521)**	**(n = 321)**
Sex, % female (n)	83.3 (434)	72.0 (231)*
Age at inclusion, years (range)	51.6 ± 11.2 (19 to 80)	39.4 ± 15.1 (18 to 78)*
Age of onset, years (range)	40.7 ± 12.8 (2 to 75)	NA
Duration of disease, years (range)	10.8 ± 8.3 (1 to 45)	NA
Rheumatoid factor, IU/ml^a^	149.8 ± 67.2	ND
Rheumatoid factor-positive, % (n)	55.0 (279)	ND.
Anti-CCP antibody, RU/ml^b^	67.5 ± 53.7	ND
Anti-CCP positive, % (n)^c^	78.6 (239)	ND
C-reactive protein, mg/l	19.6 ± 23.7	ND

Values denote mean ± standard deviation unless indicated otherwise. *Significant (*P* <0.001); ^a^rheumatoid factor serum levels were determined in 507 rheumatoid arthritis cases; ^b^anti-CCP antibody serum levels were determined in n = 304 RA patients; ^c^values <4.2 RU/ml were considered as anti-CCP negative. NA, not applicable; ND, not determined; anti-CCP: antibody against cyclic citrullinated peptide specific for rheumatoid arthritis.

Synovial fibroblasts were isolated from 40 patients with longstanding RA fulfilling the American College of Rheumatology (formerly, the American Rheumatism Association) revised criteria for RA [33]. These patients underwent elective knee joint replacement surgery. We obtained written consent of patients according to the current Declaration of Helsinki. The study was approved by the Ethics Committee of the National Institute of Rheumatic Diseases, Piestany, Slovakia, and the Ethics Committee of the University of Regensburg, Germany.

### Marker selection and genetic analyses in case-control study

SNPs in *CYB5A* genomic region (NM_148923.3) with 10 kb on the 5′ and 3′ end, respectively (chromosome 18: 71,910,527-71,969,251; NCBI build 37.3), were selected based on published information from two GWAS on RA with *P* <0.01 (see Table 2, and previous publications [31,32]).

**Table 2:** ***CYB5A*** **single nucleotide polymorphism (SNP) marker used in analysis**

**SNP**	**Position on chromosome 18** ^**a**^	**Localization** ^**b**^	**Major allele (1)**	**Minor allele (2)**	***P*** **(odds ratio) in GWAS**	**TaqMan assay**	**Call rate in 842 samples**
rs1790858	71924819	Intron 3	C	T	0.0095 (0.44)^c^	C___7536288_10	1
rs1790834	71948257	Intron 1	G	A	0.0073 (0.83)^d^	C___7536393_20	0.962

^a^NCBI build 37.3; ^b^relative to NM_148923.3; ^c^previous publication [32]; ^d^previous publication [31]. GWAS, genome-wide association studies.

Genomic DNA was isolated from whole blood samples using the PureGene DNA Blood Kit (QIAGEN, Hilden, Germany). DNA samples were genotyped using 5′ exonuclease TaqMan® technology (Life Technologies, Applied Biosystems, Foster City, CA, USA) following the manufacturer’s instructions. In brief, for each genotyping experiment 10 ng DNA was used in a total volume of 5 μl containing 1 × TaqMan® Genotyping Master Mix (Applied Biosystems). Real-time polymerase chain reaction (RT-PCR) and post-RT-PCR endpoint plate read was carried out according to the manufacturer’s instructions using the Applied Biosystems 7900HT RT-PCR System. Sequence Detection System software version 2.4.1 (Applied Biosystems) was used to assign genotypes applying the allelic discrimination test. Case and control DNA were genotyped together on the same plates with duplicates of samples (10%) to assess intraplate and interplate genotype quality. No genotyping discrepancies were detected. Assignment of genotypes was performed by a person without knowledge of sample case-control status.

### Preparation of synovial tissue and culture of synovial fibroblasts

Synovial tissue samples from patients with RA were obtained immediately after opening the knee joint capsule as previously described [34]. Pieces of synovial tissue of up to 9 cm^2^ were excised. One part of the tissue sample was cut, paraffin-embedded, and stored until further use (immunohistochemistry, see below). Another part of the synovial tissue specimen was minced and put in Dispase I (Roche Diagnostics, Mannheim, Germany). Digestion lasted for at least 1 hour at 37°C on a shaking platform. The resulting suspension was filtered (70 μm filter) and spun at 300 *g* for 10 minutes. We treated the pellet with erythrocyte lysis buffer (20.7 gm NH_4_Cl, 1.97 gm NH_4_HCO_3_, 0.09 gm EDTA, and 1 liter of H_2_O) for 5 minutes followed by centrifugation for 10 minutes at 300 *g*. The pellet was resuspended in RPMI 1640 (Sigma-Aldrich, St Louis, MO, USA) with 10% fetal calf serum. We transferred 1,000,000 cells to a 75-cm^2^ tissue culture flask, incubated them overnight, and supplemented these cells with fresh medium.

Synovial fibroblasts derived from these cultures were routinely used in passages two or three, where these cells are known to conserve their phenotype [35]. Cells were seeded into 6-well plates for steroid conversion experiments or 25-cm^2^ tissue culture flasks for nucleic acid isolation and were kept in a humidified atmosphere with 5% CO_2_ at a temperature of 37°C.

### Nucleic acid extraction and quantitative RT-PCR analysis

Synovial fibroblasts were disrupted using QIAshredder colums (QIAGEN). Total cellular RNA and DNA were isolated in parallel from cultured cells using the AllPrep DNA/RNA Mini Kit (QIAGEN). Genotyping was done as described above and cDNAs were generated from RNA by reverse transcriptase reaction using AffinityScript quantitative RT-PCR synthesis kit (Agilent Technologies, Böblingen, Germany). RT-PCR was performed using TaqMan primer/probes (Applied Biosystems - Life Technologies, Darmstadt, Germany) targeting two isoforms of *CYB5A* gene (Hs00157217_m1 and Hs01076969_m1). In addition, expression of several genes coding for enzymes relevant for steroid metabolism was assessed in synovial fibroblasts using TaqMan assays: 17α-hydroxylase/17,20-lyase (Hs01124136_m1 for *CYP17A1*), NADPH-cytochrome P450 reductase (Hs01016332_m1 for *POR*), 3β-hydroxysteroid dehydrogenase isoforms (Hs00426435_m1 for *HSD3B1*, Hs00605123_m1 for *HSD3B2*, and Hs00228639_m1 for *HSD3B7*, respectively), the 25-hydroxycholesterol 7α-hydroxylase cytochrome P450 7B1 (Hs00191385_m1for *CYP7B1*), and aromatase cytochrome P450 19A1 (Hs00903413_m1 for *CYP19A1*). Relative gene expression was normalized to *HPRT1* mRNA (Hs01003267_m1) levels using the comparative cycle threshold (Ct) method and presented as expression ratio using 2^-ΔΔCt^ [36]. RA cDNA with ΔCt value next to the median of the ΔCt distribution was used as calibrator sample.

### Immunocytochemistry

Human cytochrome b5 type A was detected and quantified using immunohistochemistry. Five-micrometer sections were cut from paraffin-embedded blocks. For immunostaining, the sections were deparaffinized in xylol and ethanol and rehydrated in a descending ethanol series. The slides were placed in a 0.1 mol/L citrate buffer (pH = 6.0) and boiled for 20 minutes at 120°C. Slides were then washed in PBS buffer (pH = 7.6), and treated briefly with 2% hydrogen peroxide in 1 × PBS for 10 minutes to block endogenous peroxidase activity. Slides were incubated for 45 minutes in blocking solution consisting of 10% bovine serum albumin (PAA, Inc., Pasching, Austria), 10% fetal calf serum (PAA, Inc.), and 10% goat serum (Sigma, Steinheim, Germany). After blocking, sections were incubated with primary antibody (1:500, ab69801, Abcam, Cambridge, MA) overnight at room temperature. After incubation with biotinylated secondary antibody (1:200, polyclonal goat anti-rabbit IgG biotinylated, from Dako, Hamburg, Germany) for 90 minutes, the sections were treated with streptavidin-horseradish peroxidase (GE Healthcare Europe GmbH, Munich, Germany). Visualization was performed with diaminobenzidine tetrahydrochloride hydrate (ImmPACT DAB substrate, Vector Laboratories, Peterborough, UK). Staining with control serum always yielded negative results, and liver tissue was used as positive control. In order to determine the tissue density of cytochrome b5-positive cells, the number of positive cells was averaged from 17 randomly selected high-power fields (400×). The number of investigated high-power fields was derived from a pioneering histological study [37].

### Incubation with radiolabeled steroids and steroid extraction

For steroid conversion experiments synovial fibroblasts in 6-well plates were washed with PBS and incubated with RPMI 1640 (without phenol red or serum, but supplemented with 15 mM HEPES, 2 mM stable glutamine and 40 mg/l gentamycin) (PAN Biotech, Aidenbach, Germany). After 3 hours, the radiolabeled substrate 7-^3^H(N)-pregnenolone (Preg, 5-pregnen-3β-ol-20-one) (PerkinElmer, Rodgau-Jügesheim, Germany) was added for another 24 hours at a final concentration of 25 nM containing an activity of 37,000 Bq.

The fibroblast monolayers were fixed overnight with 10% formaldehyde, washed with saline, and nuclei were stained with Hoechst 33258. Large-area images were taken on a Cell Observer microscopic system (Carl Zeiss, Jena, Germany) and counted nuclei were taken as cell numbers for normalization of steroid conversion results.

From the cell-free supernatants steroids were extracted twice with 3 ml cold ethyl acetate. The exact concentration of radiolabeled steroid applied to every well and the extraction efficiencies were monitored by liquid scintillation counting of aliquots in Ultima Gold cocktail (PerkinElmer): recoveries of radioactivity in the organic phase varied insignificantly (97.2 ± 1.5%, mean ± SD). The stable extracts were lyophilized in a speed-vac concentrator (Saur, Reutlingen, Germany) and stored at −20°C until analysis.

### Two-dimensional thin-layer chromatography (2D-TLC) of steroids

**Figure 1:**
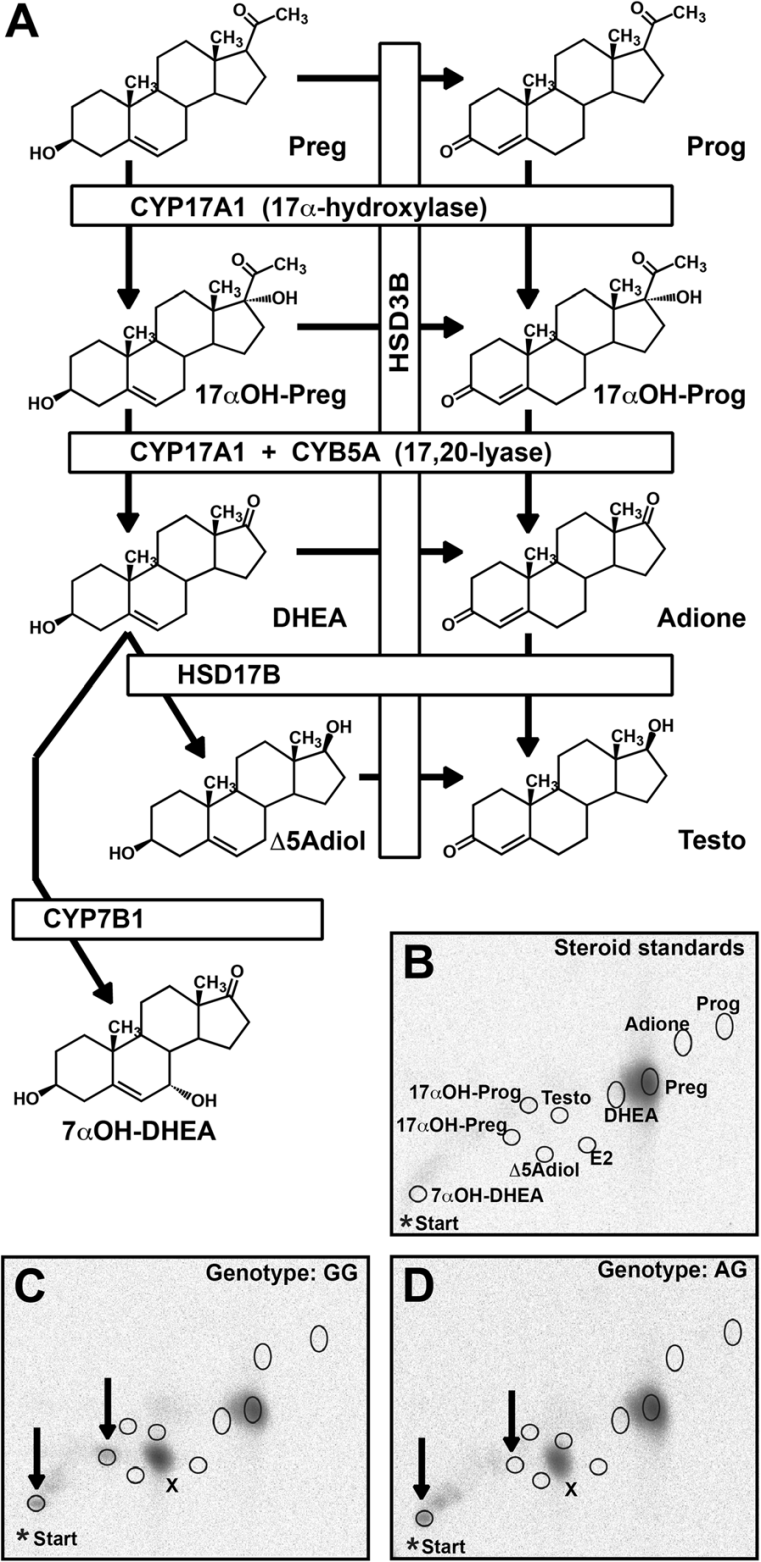
**Analysis of pregnenolone metabolism in rheumatoid arthritis synovial fibroblasts.** **(A)** Pathways and enzymes of pregnenolone metabolism as identified in synovial fibroblasts (reviewed in a previous publication [29]). **(B,** **D)** Two-dimensional thin layer chromatography (2D-TLC) separation of pregnenolone and its metabolites. **(B)** Position of unlabeled steroid standards of identified metabolites (circles) overlaid to the ^3^H-image of the pregnenolone substrate used. **(C)** ^3^H-image from a GG-fibroblast line accumulating (besides compound X) 17α-hydroxy-pregnenolone, the direct product 17α-hydroxylase activity, together with 7α-hydroxy-dehydroepiandrosterone (DHEA). **(D)** ^3^H-image from an AG-fibroblast line accumulating almost exclusively 7α-hydroxy-DHEA. *CYB5A*, cytochrome b5, type A; *CYP17A1*, steroid 17α-hydroxylase/17,20-lyase (cytochrome P450 17A1). E2 was found in a minority of fibroblasts only. *Start indicates point of sample application. X indicates the major product formed from pregnenolone by fibroblasts independently from rs1790834 genotype. Arrows indicate the metabolites with production dependent on the rs1790834 genotype. *CYP7B1*, 25-hydroxycholesterol 7α-hydroxylase (cytochrome P450 7B1); *HSD3B*, 3β-hydroxysteroid dehydrogenase; Δ5Adiol, Δ5-androstenediol; Adione, androstenedione; Preg, pregnenolone; Prog, progesterone; Testo, testosterone. E2, estradiol.

Pregnenolone and its metabolites with respect to androgen synthesis [29] (Figure 1A) were separated essentially as described previously [22], with modifications as given below. Solvents and other reagents were purchased from Merck (Darmstadt, Germany), if not stated otherwise. Unlabeled steroids were from Sigma (Steinheim, Germany) and from Steraloids (Newport, RI, USA). Stock solutions were prepared in ethanol. Lyophilized steroid extracts were dissolved in 50 μl ethanol, spotted under a nitrogen stream with a Linomat IV (Camag, Muttenz, Switzerland) on silica gel 60 F254 TLC aluminum sheets (Merck) together with a mixture of unlabeled carrier steroids (Figure 1B). The first separation of 2D-TLC was done in toluene:methanol (90:10). The second dimension of the separation was done by two successive developments in chloroform:diethylether (90:10). For identification of spots, the 2D-TLC plates were stained with copper acetate in phosphoric acid as described previously [22]. Radioactivity on the 2D-TLC plates was quantified by radioimaging on an FLA 3000 (Fuji-Raytest, Straubenhardt, Germany). Spots were assigned only if their intensity was more than two standard deviations above background. Spot intensities were corrected for corresponding values obtained from blank wells incubated with radiolabeled pregnenolone without cells. The results were calculated as pmol of steroid produced per million cells in a 24-hour incubation period.

### Statistical and bioinformatical analyses

Differences between dichotomous traits were calculated employing the chi-square (*χ*^2^) test. Differences in continuous variables between groups were calculated using the two-tailed *t*-test for normally distributed data or the non-parametric Mann-Whitney *U*-test for variables not normally distributed as determined by the Shapiro-Wilk test. Expression data were compared between the three genotypes after grouping the minor allele carriers (dominant model). Logistic regression was used for the different models in association analyses (sex as a covariate, stratified by sex, and stratified by either RF or anti-CCP status). Odds ratios (OR) and 95% CI were calculated. To determine whether the genotypes of cases and controls of all SNPs deviated from Hardy-Weinberg equilibrium, actual and predicted genotype counts of both groups were compared by the exact test [38]. A *P*-value <0.05 was considered significant.

Association analyses were performed with PLINK v1.07 [39]. Statistical software packages JMP 7.0.2 (SAS Institute Inc, Cary, NC, USA) and SigmaPlot 11.2.05 Systat Software Inc, San Jose, CA, USA) were used for other analyses. Meta-analysis weighted according to sample size was performed using METAL with Cochran’s *Q*-test for heterogeneity [40,41]. For linkage disequilibrium testing, HaploView v4.2 was employed [42] with HapMap version 3 data from release 28 [43]. A power calculation was performed with Quanto 1.2.4 (Division of Biostatistrics, USC University of Southern California, Los Angeles, CA, USA) assuming a population risk of 1% for RA. MatInspector v8.05 and SNPInspector 2.2 (Genomatix Software GmbH, Munich, Germany) were used to analyze the potential functional consequences of SNPs on transcription factor binding sites.

Gene expression and steroid conversion data are shown as mean ± SD, if all data in a figure were normally distributed. Otherwise, data are presented as box plots, where boxes represent the 25th and 75th percentiles, respectively. The median is indicated by a horizontal line. The whiskers indicate the 10th and 90th percentiles, respectively. Dots represent individual outliers.

## Results

### Genotyping and association study

Two recently published GWAS on RA susceptibility [31,32] were screened for association between disease and *CYB5A* polymorphisms, resulting in two SNPs with *P* <0.01. In our Slovak RA case-control sample (Table 1), we sought direct replication of these SNPs (Table 2). In our sample, controls were significantly younger than cases at inclusion into the study but were in a comparable age range when the onset of disease in RA cases was compared to age at inclusion of controls. Significantly more females were present in the case group. Both markers fulfilled our criteria of at least a 95% call rate and no deviation from Hardy-Weinberg equilibrium was observed (Table 3). Allele frequencies for the two SNPs were consistent between HapMap release 28 data for the population of Utah residents with ancestry from northern and western Europe (called the CEU population) and our disease-free controls (1% and 18% in HapMap and 1.2% and 18.9% in controls for rs1790858 and rs1790834, respectively).

**Table 3:** ***CYB5A*** **single nucleotide polymorphism (SNP) association analysis results in a rheumatoid arthritis (RA) case-control replication sample of Slovak patients and controls**

	**RA case genotypes**					**RA-free control genotypes**					**Association results**	
**SNP**	**11**	**12**	**22**	**MAF**	***P*** **(HWE)**	**11**	**12**	**22**	**MAF**	***P*** **(HWE)**	***P*** ^**a**^	**Odds ratio for minor allele (95% CI)** ^**a**^
rs1790858	510	11	0	0.011	1	313	8	0	0.012	1	0.835	0.91 (0.36, 2.30)
rs1790834	388	122	11	0.138	0.71	187	95	7	0.189	0.25	8.5*10^−3^	0.69 (0.52, -0.91)

^a^Logistic regression adjusted for sex. Numbers of genotypes according to alleles from Table 2. MAF, minor allele frequency; HWE, Hardy-Weinberg equilibrium.

After adjustment for sex, rs1790834 showed association with risk of RA (*P* = 8.5*10^−3^; OR = 0.69, 95% CI 0.52, 0.91) (Table 3). Additional adjustment for age (age at inclusion for controls and age of onset for RA patients) did not change the association results (data not shown). Meta-analysis with the initial association data from a previous publication [31] resulted in *P* = 3.6*10^−4^ (OR = 0.76, 95% CI 0.69, 0.84), that is, a protective effect of the minor allele. No heterogeneity was detected (*P* = 0.237).

When the association of rs1790834 with risk of RA was analyzed separately for both sexes, there was significant association only for women with *P* = 4.1*10^−3^; OR = 0.63 (95% CI 0.46, 0.86; n = 434 cases, n = 231 controls). There was no association detectable for men (n = 87 cases, n = 90 controls) in our study sample (*P* = 0.81; OR = 0.93, 95% CI 0.52, 1.67).

In addition, stratified analysis for RF-positive (n = 279) and RF-negative cases (n = 228) was performed: rs1790834 showed only significant association results in RF-positive cases with *P* = 3.3*10^−4^ (OR = 0.53, 95% CI 0.37, 0.75) in contrast to RF-negative cases with *P* = 0.38 (OR = 0.86, 95% CI 0.62, 1.20). In addition, in the anti-CCP positive subset of RA patients, significant association between rs1790834 and the disease was detected with *P* = 2.7*10^−3^ (OR = 0.58, 95% CI 0.41, 0.83).

### Gene expression analysis

**Figure 2:**
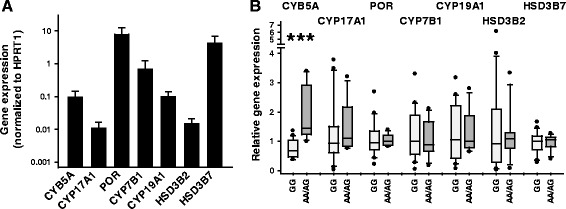
**Effect of rs1790834 genotypes on expression of** ***CYB5A*** **and steroidogenic genes.** Real-time quantitative PCR analysis of mRNA expression in 22 RA synovial fibroblast lines (rs1790834 genotype GG, n = 15; AA/AG genotypes, n = 7). **(A)** Synovial fibroblasts express all genes, which are necessary for synthesis of biologically active androgens. Mean ± SD of expression levels normalized to *HPRT1* of combined data for all genotypes. **(B)** rs1790834 genotype-dependent expression levels, which are normalized to the median value of the combined expression data for each gene. In the box-and-whisker plots the genotypes with the rare allele A were grouped together (AA, n = 1; AG, n = 6). The boxes represent the 25th to 75th percentiles, and horizontal lines within the box represent median values. The whiskers show the 10th and 90th percentiles, respectively, and dots represent individual values exceeding these limits. ***Significant rs1790834 genotype-dependent expression, *P* <0.005, Mann-Whitney rank-sum test. *CYB5A*, cytochrome b5, type A; *CYP17A1*, steroid 17α-hydroxylase/17,20-lyase (cytochrome P450 17A1); *CYP19A1*, aromatase (cytochrome P450 19A1); *CYP7B1*, 25-hydroxycholesterol 7α-hydroxylase (cytochrome P450 7B1); *HPRT1*, hypoxanthine(−guanine) phosphoribosyltransferase 1; *HSD3B2/7*, 3β-hydroxysteroid dehydrogenase isoforms 2/7; *POR*, NADPH-cytochrome P450 reductase.

Synovial fibroblast RNA samples from 22 RA patients with measured rs1790834 genotypes were available for gene expression analysis. SNP rs1790834 is located in the first intron of the *CYB5A* gene, that is, not within the protein coding sequence. Therefore, we first verified expression of *CYB5A* and several key genes of pregnenolone metabolism: fibroblasts expressed the longest mRNA-transcript of *CYB5A* (GenBank NM_148923; cytochrome b5 isoform 1), which encodes the membrane-bound fully functional cytochrome b5. In addition, *CYP17A1* and *POR* as prerequisite for DHEA synthesis (and hence androgen synthesis), and *HSD3B2*, *HSD3B7*, *CYP7B1* and *CYP19A1* as prerequisite for downstream processing of DHEA were expressed in synovial fibroblasts (Figure 2A), whereas *HSD3B1* was not found.

In a second step, we tested whether the minor rs1790834 A-allele has an effect on gene expression levels. Comparing *CYB5A* mRNA expression levels, the mean expression of *CYB5A* was significantly higher within carriers of the minor allele (Figure 2B). The rs1790834 genotype had no effect on the expression levels of the other steroidogenic genes expressed in synovial fibroblasts (Figure 2B), indicating that the SNP specifically affects *CYB5A* expression.

Biobank tissue sections were available for a subgroup of RA patients. In these tissue sections, we studied immunohistochemically investigated density of cytochrome b5-positive cells. Density of positive cells positively correlated with amount of mRNA (n = 8, *r*^2^ = 0.64, *P* = 0.017; data not shown). Thus, the minor rs1790834 A-allele facilitates higher cellular cytochrome b5 contents.

### *CYB5A* genotype affects pregnenolone metabolism in RA synovial fibroblasts

As fibroblasts metabolize various steroid substrates, we tested whether the *CYB5A* genotype affects the conversion of pregnenolone into downstream metabolites. This pathway was studied because the key enzyme for androgen synthesis, the 17,20-lyase activity of cytochrome P450 17A1, depends on cytochrome b5, the product of the *CYB5A* gene.

**Figure 3:**
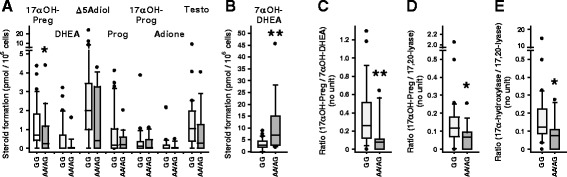
***CYB5A*** **genotype affects pregnenolone metabolism in rheumatoid arthritis (RA) synovial fibroblasts.** **(A,** **B)** Products of pregnenolone metabolism in 40 synovial fibroblast lines from RA patients genotyped for rs1790834 (n = 27, GG genotype; n = 13, genotypes AA/AG). **(C,** **E)** Product ratios indicate that the *CYB5A* A allele increases steroid 17,20-lyase activity of *CYP17A1* gene product. Shown are the calculated ratios 17α-hydroxy-pregnenolone/7α-hydroxy- dehydroepiandrosterone (DHEA) **(C)**, 17α-hydroxy-pregnenolone/17,20-lyase **(D)**, and 17α-hydroxylase/17,20-lyase **(E)**, where 17,20-lyase refers to the sum of all C19-steroids produced and 17α-hydroxylase refers to the sum of 17α-hydroxy-pregnenolone plus 17α-hydroxy-Prog. Significant differences in rs1790834 genotype-dependent metabolite levels or product ratios, respectively, were identified with the Mann-Whitney test: *P <0.05; **P <0.01. Δ5Adiol, Δ5-androstenediol; Adione, androstenedione; Preg, pregnenolone; Prog, progesterone; Testo, testosterone.

Synovial fibroblasts from 40 genotyped RA patients (n = 27 for major allele GG carriers, n = 13 for minor allele AA/AG carriers) converted radiolabeled pregnenolone into a variety of steroids (for products/pathways identified see Figure 1A). By 2D-TLC we identified nine products of pregnenolone metabolism (Figure 1B-D). There were no allele-dependent differences in the production of progesterone, 17α-hydroxyprogesterone, DHEA, androstenedione, androstenediol, testosterone, and an unidentified product X, which was the major product from fibroblasts, but independent of genotype (GG: 187 ± 152 versus AA/AG: 233 ± 175 pmol/(10^6^ cells*24 hours), mean ± SD; *t*-test: *P* = 0.40) (Figure 3A, B).

Accumulation of the direct product of the 17α-hydroxylase activity, 17α-hydroxy-pregnenolone, in synovial fibroblasts from carriers of the minor A-allele was significantly lower than in non-carriers (GG-genotype) (Figure 3A). Carriers of the minor A-allele produced roughly 2.5 times the amount of 7α-hydroxy-DHEA than non-carriers (*P* = 0.009, Figure 3B). As production of 7α-hydroxy-DHEA depends on DHEA availability this metabolite indicates increased 17α-hydroxylase and 17,20-lyase activity.

Decreased ratios of 17α-hydroxylase over 17,20-lyase activity would be expected when the *CYB5A* A-allele stimulates the latter enzyme step via increased availability of cytochrome b5. Therefore, metabolite ratios were calculated: 17α-hydroxy-pregnenolone/7α-hydroxy-DHEA, 17α-hydroxy-pregnenolone/17,20-lyase (that is, the sum of all C19-steroids produced), and 17α-hydroxylase (that is, 17α-hydroxy-pregnenolone plus 17α-hydroxy-progesterone)/17,20-lyase, were significantly decreased (*P* = 0.006, *P* = 0.022, and *P* = 0.03, respectively) in synovial fibroblasts from RA patients with the rs1790834 A-allele (Figure 3C-E), clearly indicating an effect of the *CYB5A* genotype on steroid 17,20-lyase activity.

Finally, the two groups of the study were compared in order to check homogeneity with respect to clinical variables. The patients did not receive intra-articular glucocorticoid therapy. Importantly, age, sex, and prednisolone dosage was similar in the group with the minor allele (A) as compared to the group with the major allele (G) (age 63 versus 68 years; female/male 83%/17% versus 90%/10%; and dosage mean ± standard error of the mean 4.58 ± 1.00 versus 6.50 ± 1.50 mg/day). Similarly, the frequency of prednisolone and methotrexate treatment was similar in the two groups (prednisolone 83% versus 80%, and methotrexate 33% versus 10%). The patients did not receive anti-TNF therapy or other disease-modifying anti-inflammatory drugs because the disease was in a mild and late stage. In addition, markers of inflammation like C-reactive protein (23.4 ± 14.8 versus 16.8 ± 4.1 mg/l) and erythrocyte sedimentation rate (21 ± 5 versus 39 ± 17 mm/1st hour) were not significantly different between groups.

## Discussion

Owing to the design of GWAS, below their threshold of unambiguous detection important genetic variants remain hidden (in a so-called sea of noise). Nevertheless, these genetic variants may contribute significantly to the risk of RA. Instead of enforcing more statistical power by increasing numbers of cases and controls in GWAS, a hypothesis-driven approach combined with functional analyses can help to unravel significant contributions of candidate genes to risk of RA [44]. RA is associated with comparatively low androgen levels, both systemically and within the inflamed tissue [20,24,25,45,46]. Therefore, we screened the data sets of two published GWAS for polymorphisms in the *CYB5A* gene [31,32], which is relevant for steroid metabolism and identified two RA-associated SNPs linked to low androgen secretion.

In our Slovak case-control study sample, the minor allele of SNP rs1790834 was associated with reduced risk for RA. The power for this replication of a GWAS finding [31] was about 42% in our study with 521 cases and 321 controls (using the reported OR = 0.83, minor allele frequency = 18% and one-sided *P*-value = 0.05). In subgroup analysis, we detected association between SNP rs1790834 in RF-positive RA cases only. This is consistent with the initial study by Plenge *et al*., including patients that were seropositive for anti-CCP [31]. Anti-CCP is a better diagnostic marker than RF, and the correlation between the two factors is moderate [47]. Although anti-CCP levels were only available in a subset of our RA patients, we again found a strong association between rs1790834 and the disease in this subgroup. Altogether, our data and the initial data by Plenge *et al*. [31] suggest that rs1790834-driven association with RA is limited to an RF/anti-CCP-positive disease entity. Heritability is in a similar range for both anti-CCP-positive and -negative RA, but these represent different disease entities [48].

Importantly, stratification for sex revealed an association between rs1790834 and RA only in women in our study, indicating a strong sex bias for the protective effect of the minor allele A. One possible explanation is the reduced power in our male sample with 87 cases and 90 controls (36% power given an OR of 0.69, an allele frequency of 18.9% in controls, a nominal significance with one-sided *P*-value = 0.05 in an additive model and an assumed population risk of 1%). In comparison, the power was 77% to detect this effect in women (434 cases and 231 controls). However, as RA is a disease with higher prevalence in women than in men, sexual hormones are thought to possibly be involved in disease onset and progression [4].

With a minor allele frequency of about 18% for rs1790834 this *CYB5A* polymorphism is not rare. Additional SNPs may even increase the contribution of *CYB5A* polymorphisms to reduction of RA risk. Our inability to replicate the effect of the other SNP, rs1790858 [32], does not contradict this notion because the protective allele frequency of 1% for this SNP is too low to be detected by our study design. In contrast, it suggests that in future work all independent SNPs in the *CYB5A* gene should be analyzed in detail for their possible contribution to RA risk.

Cytochrome b5, the protein encoded by the *CYB5A* gene, has diverse functions, which are of varying importance in different tissues and cell types. Notably, NADH-dependent reduction of methemoglobin (erythrocytes), electron transfer to fatty-acid desaturases (mainly liver), and steroid and xenobiotics metabolism by cytochrome P450 enzymes depend on cytochrome b5 [29,49,50]. Cytochrome P enzyme activity is increased by at least two different mechanisms: either cytochrome b5 mediates electron transport to cytochrome P450 enzymes or it facilitates allosteric interaction of selected cytochrome P450 enzymes with the electron donating NADPH-cytochrome P450 reductase [49,50]. The latter mechanism potentiates the activity of the affected cytochrome P450 enzymes, amongst them enzymes involved in metabolism of xenobiotics [49], and the key enzyme activity for androgen biosynthesis, cytochrome P450 17A1 steroid 17,20-lyase, our target enzyme [30]. As androgens and androgen metabolism play a role in the etiology of RA, there was an obvious need to further analyze the SNP rs1790834 in this context.

To test for functional consequences of *CYB5A* polymorphisms, a patient-specific source of tissue or cells was indispensable. With respect to androgen synthesis, this would be adrenal glands or gonads [29,51], which are unavailable for these experimental studies in RA. On the other hand, synovial fibroblasts are a validated model for gene expression and functional studies in RA research. They maintain a well-conserved phenotype in cell culture over several passages [35,52], and patient-derived fibroblast lines largely maintain their gene expression profiles [35].

We consistently detected significant amounts of pregnenolone converting enzymes in synovial fibroblasts. As expected for a cell type that is not primarily involved in androgen production, the activities of those enzymes are low when compared to activities in adrenals or gonads [29,30,51]. Nevertheless, we found all enzymes necessary for and all metabolites indicative of androgen biosynthesis in synovial fibroblasts. Moreover, the critical step of androgen synthesis, the 17,20-lyase activity of *CYP17A1*-encoded cytochrome P450 17A1, clearly depends on the *CYB5A* alleles (determining the amount of cytochrome b5). High levels of cytochrome b5 can increase androgen synthesis more than 10-fold in experiments using purified enzymes from adrenals or gonads, respectively, or using recombinant enzymes [30,53]. We found a 2- to 3-fold increase of the capacity for androgen synthesis in synovial fibroblasts harboring the RA-protective allele A of SNP rs1790834, which parallels the increase in *CYB5A* expression. This effect size is reasonable, as (1) almost all tested fibroblast lines were from heterozygous donors (AG) and (2) the major allele homozygous carriers (GG) express *CYB5A* (at normal levels). Neither the expression levels of *CYP17A1* nor that of any other gene analyzed were altered. Therefore, the intronic SNP, which does not alter the protein sequence of the *CYB5A*-encoded cytochrome b5, most likely exerts its effect specifically via control of the expression level.

It should be emphasized that the accumulation of (proinflammatory) 7α-hydroxy-DHEA is a specific feature of synovial fibroblasts, which express high levels of *CYP7B1*-encoded cytochrome P450 7B1 [26,27], but not a consequence of cytochrome b5 levels in general. For tissues or cell populations, where cytochrome P450 7B1 is not the dominant enzyme for DHEA metabolism, the *CYB5A* SNP would modulate the production of other androgens and their metabolites.

Although adrenals and gonads from RA patients or healthy controls are not amenable to direct testing, our results implicate that the identified *CYB5A* SNP may be generally responsible for facilitated androgen production in these tissues of minor allele carriers. Correspondingly, the major allele determines lower androgen levels [54], which precede disease onset in RA patients [15,16]. In addition, local androgen production in the inflamed joints would be hampered by the major allele after disease onset [20,24,25].

In summary, the identified *CYB5A* SNP should affect the 17,20-lyase step of androgen biosynthesis in all tissues expressing cytochrome P450 17A1. Due to the modulatory role of cytochrome b5 on 17,20-lyase activity, as outlined above, the alterations in pregnenolone metabolism resulting from the possible SNP variant combinations are expected to be moderate. They should cause less dramatic changes of the androgen (and downstream metabolite) concentrations, than found, for example, in human isolated 17,20-lyase deficiency [55] or in the cytochrome b5 knockout mouse [56], where androgens are massively depleted. Nevertheless, the *CYB5A* SNP surely contributes to the variance in androgen levels in the population, which will be inconspicuous in the majority of individuals. However, if activities of other enzymes involved in steroid metabolism are low, the rs1790834 (GG) carriers are more likely than carriers of the A allele to present signs of partial gonadal or adrenal failure [20,28,29,51,57,58]. This was suggested to be involved in the etiology of RA [15,16].

## Conclusions

The *CYB5A* SNP contributes to the heritable risk in RA women. It can be an important factor contributing to the sex bias in RA incidence. As androgen levels in women are generally lower than in men, any additional decrease will disproportionately reduce the anti-inflammatory impact of androgens. In other words, the risk-lowering minor allele of SNP rs1790834 may help to ensure protective androgen levels in women, whereas the major allele may contribute to low androgen levels, which are insufficient to block development of RA or to ameliorate the inflammatory processes. In this setting the *CYB5A* SNP rs1790834 is rather unimportant in men, which is reflected by the lack of a protective role of the risk-lowering minor allele of SNP rs1790834 in the male subgroup. A clinical implication of our results lies in the fact that any kind of androgen therapy can be successful preferentially in *CYB5A* SNP rs1790834 major allele carriers.

## Abbreviations

2D-TLC: two-dimensional thin-layer chromatography
anti-CCP: antibody against cyclic citrullinated peptide specific for rheumatoid arthritis
Ct: cycle threshold
DHEA: dehydroepiandrosterone
ELISA: enzyme-linked immunosorbent assay
GWAS: genome-wide association studies
IL: interleukin
OR: odds ratio
PBS: phosphate-buffered saline
POR: NADPH-cytochrome P450 reductase
RA: rheumatoid arthritis
RF: rheumatoid factor
RT-PCR: real time polymerase chain reaction
SNP: single nucleotide polymorphisms
TNF: tumor necrosis factor
